# Maternal stress or sleep during pregnancy are not reflected on telomere length of newborns

**DOI:** 10.1038/s41598-020-71000-2

**Published:** 2020-08-19

**Authors:** Antti-Jussi Ämmälä, Emma I. K. Vitikainen, Iiris Hovatta, Juulia Paavonen, Outi Saarenpää-Heikkilä, Anneli Kylliäinen, Pirjo Pölkki, Tarja Porkka-Heiskanen, Tiina Paunio

**Affiliations:** 1 Department of Genetics and Biomarkers, National Institute for Health and Welfare, Mannerheimintie 166, P.O. 30, 00271 Helsinki, Finland; 2 Department of Psychiatry, University of Helsinki and Helsinki University Hospital, Helsinki, Finland; 3 Organismal and Evolutionary Biology Research Programme, Faculty of Biological and Environmental Sciences, University of Helsinki, Helsinki, Finland; 4 Department of Psychology and Logopedics, University of Helsinki, Helsinki, Finland; 5 SleepWell Research Program, Faculty of Medicine, University of Helsinki, Helsinki, Finland; 6 Neuroscience Center, Helsinki Institute of Life Science HiLIFE, University of Helsinki, Helsinki, Finland; 7 Department of Public Health Solutions, National Institute for Health and Welfare, Helsinki, Finland; 8 Pediatric Research Center, Child Psychiatry, University of Helsinki and Helsinki University Hospital, Helsinki, Finland; 9 Department of Paediatrics, Tampere University Hospital, Tampere, Finland; 10 Tampere Centre for Child Health Research, Tampere University, Tampere, Finland; 11 Psychology, Faculty of Social Sciences, Tampere University, Tampere, Finland; 12 Department of Social Sciences, University of Eastern Finland, Kuopio, Finland

**Keywords:** Behavioural genetics, Molecular medicine

## Abstract

Telomeres play an important role in maintaining chromosomal integrity. With each cell division, telomeres are shortened and leukocyte telomere length (LTL) has therefore been considered a marker for biological age. LTL is associated with various lifetime stressors and health-related outcomes. Transgenerational effects have been implicated in newborns, with maternal stress, depression, and anxiety predicting shorter telomere length at birth, possibly reflecting the intrauterine growth environment. Previous studies, with relatively small sample sizes, have reported an effect of maternal stress, BMI, and depression during pregnancy on the LTL of newborns. Here, we attempted to replicate previous findings on prenatal stress and newborn LTL in a sample of 1405 infants using a qPCR-based method. In addition, previous research has been expanded by studying the relationship between maternal sleep quality and LTL. Maternal prenatal stress, anxiety, depression, BMI, and self-reported sleep quality were evaluated with self-reported questionnaires. Despite sufficient power to detect similar or even considerably smaller effects than those previously reported in the literature, we were unable to replicate the previous correlation between maternal stress, anxiety, depression, or sleep with LTL. We discuss several possible reasons for the discrepancies between our findings and those previously described.

## Introduction

Intrauterine growth can be seen both as a special environment and a developmental period that shapes future health and disease of a child^1^. Several maternal factors such as maternal physical, biological, psychological, and behavioural conditions can have an effect on this development via multiple channels (immunological, hormonal, and metabolic pathways). The effect of these pathways might be seen in Leukocyte Telomere length (LTL) of a child. As most cell divisions happen during the early stages of development, critical environmental factors that affect cell division and LTL may have the greatest effect during this time period. Several studies support this reasoning^1–5^. This chain of association from maternal health, LTL of a child, and child’s future health is called the Fetal Programming of Telomere Biology hypothesis^1^. It postulates that the intrauterine environment in part influences TL at birth, which in turn may regulate future development and eventually even morbidity and mortality^6,7^.

Telomeres play an important role in maintaining chromosomal integrity. Telomeres consist of repetitive DNA sequences at the end of each chromosome and a surrounding protein complex, which protects the end of the chromosome^8^. In each cell division, this sequence is shortened due to an end-replication problem and it inevitably reaches a critical point where chromosomal structure and integrity cannot no longer be maintained, resulting in a signaling cascade leading to apoptosis. Telomere length is determined by both genetic and environmental factors, often interacting with each other, making leukocyte telomere attrition both a causal and a potentiating factor for health and disease^9^. Age is a major contributor to this shortening and relative leukocyte telomere length (LTL) is thought to serve as an indicator of biological aging^5^.

In addition to chronological age, lifestyle factors such as smoking or excess alcohol consumption, and other factors such as body mass index (BMI), can also have an effect on telomere shortening^10–12^. Thus, aging is a dynamic process and reciprocal to many environmental and health-related factors. Indeed, shortened TL has previously been associated with several somatic and psychiatric diseases and conditions in adulthood^13–15^. LTL is also associated with mortality and morbidity^16–19^. Although heritability estimates for TL are as high as 70%, genetic studies are able to predict only a minor component of this variation^12,20,21^. Therefore, environmental factors clearly have a significant influence on leukocyte telomere length (LTL).

Only a few studies have explored the effect of various maternal and pregnancy-related factors on the LTL of the newborn child. Among the most studied factors is maternal stress during pregnancy^22–25^. For example, in a sample of 319 newborns, maternal stress during pregnancy was associated with shorter newborn LTL. This stress did not affect the mothers’ LTL, however, which suggests vulnerability in early stages of development^22^. Another interesting finding in the same study was that the mothers’ lifetime psychiatric morbidity correlated with the LTL of the mother, but not the offspring.

In addition to stress, an association between the maternal pre-pregnancy weight and LTL of newborns has also been shown, with shorter child LTL associated with increased pre-pregnancy weight of the mother^26,27^. Some of these findings were only observed in male offspring^26^. Similar findings were also observed between maternal education and telomere shortening^28^. Ethnicity^29^, maternal smoking^26^, gestational diabetes^30^, and maternal folate concentration^31^ and depression^26^ have also been associated with newborn LTL.

Only anecdotal knowledge exists regarding the effect of maternal sleep during pregnancy^26,27,32,33^ on newborn LTL. Disordered sleep is common during pregnancy^33,34^ and may have serious fetal consequences, such as impact on growth or other pregnancy-related outcomes. Disturbed sleep is especially common towards the end of pregnancy, such as in the third trimester^34^. We sampled 1405 newborns for LTL and tested for associations with maternal prenatal insomnia and snoring. In addition, using a dataset two to five times larger than those previously reported, we tested the robustness of previous associations between newborn LTL and maternal stress, anxiety, depression, and BMI during pregnancy.

## Results

### Analysis of correlations between prenatal variables and leukocyte telomere length of a newborn

**Figure 1:**
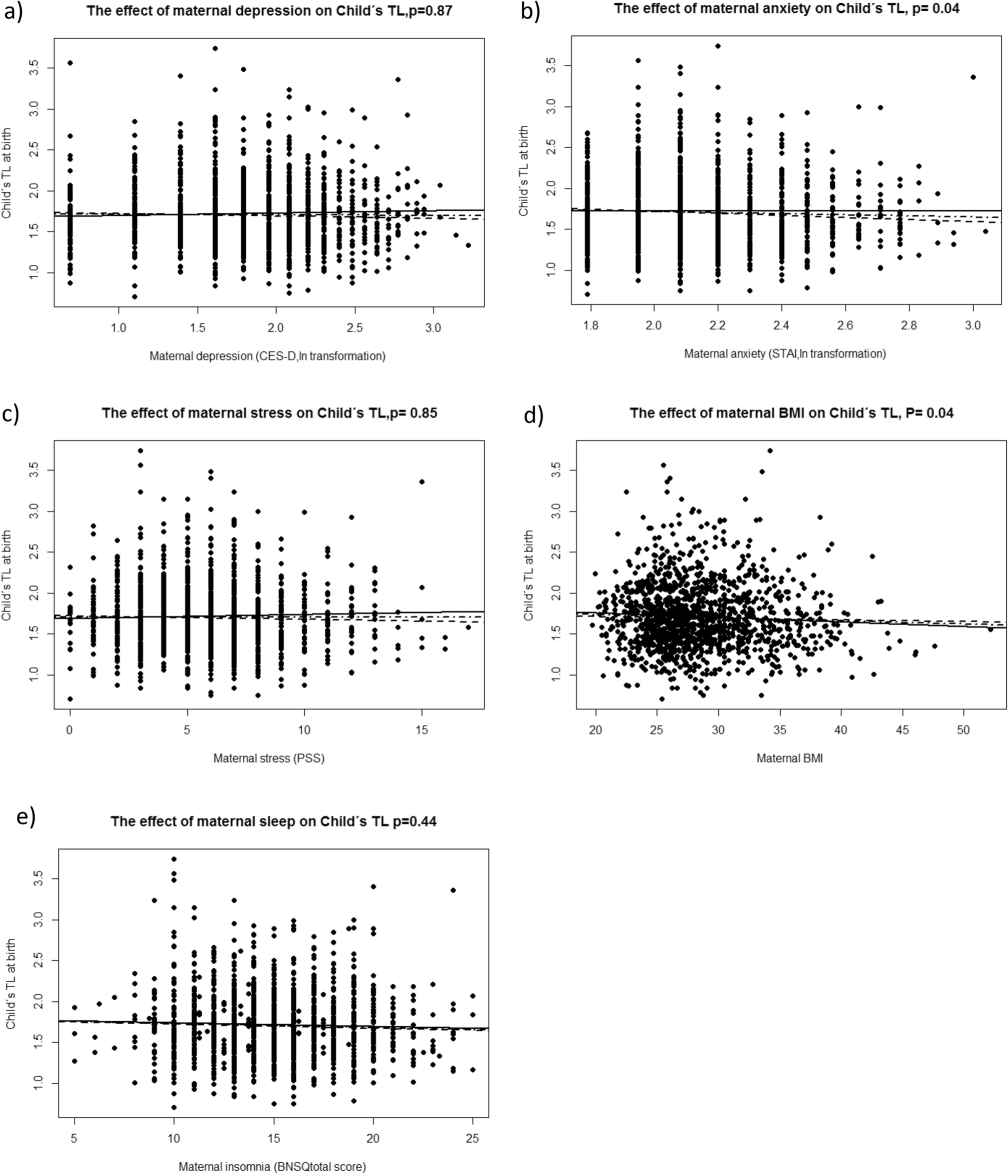
Maternal prenatal factors and their association with Child’s Telomere length at birth. (**a**) Association between maternal depression (*p* = 0.87) and child’s TL at birth. (**b**) Association between maternal anxiety (p = 0.04) and child’s TL at birth. (**c**) Association between maternal stress (*p* = 0.85) and child’s TL at birth. (**d**) Association between maternal BMI (*p* = 0.04) and child’s TL at birth. (**e**) Association between Maternal sleep (*p* = 0.65) and Child’s TL at birth. Linear regression lines: DotDashline(.-.-.-.-) = all, Dashed line(---) = Males, Solid line (___) females. BNSQ = Basic Nordic Sleep questionnaire; CES-D = Center for Epidemiological Studies Depression Scale; STAI = Short version of State and Trait anxiety Scale,Trait version;PSS = Perceived Stress Scale; BMI = Height in meters/weight^2^; LTL = relative leukocyte telomere length.

Of all explored variables, anxiety (STAI) (β − 0.09, *p* = 0.04) and BMI (β − 0.01, *p* = 0.04) had a nominal significant negative association between prenatal variables and newborn LTL (Tables 1, 2 and 3; see also Fig. 1a–e). However, neither survived the correction (FDR) for multiple testing (STAI p FDR_corr_ = 0.12, BMI pFDR_corr_ = 0.12). In most cases, the estimates were below the minimum detectable level calculated in power analysis (> 0.07).

**Table 1:** Linear regression model with Leukocyte Telomere length as a dependent variable and depression (CESD); plate; child ‘s gender; maternal smoking; maternal BMI; gestational age at birth; and maternal BNSQ total score and snoring (BNSQ16) as explanatory variables.

Unstandardized coefficients	Standardized coefficients			t	Sig	95% confidence interval for B	
	B	SE	Beta			Lower Bound	Upper Bound
(Constant)	2.37	0.38		6.19	0.00	1.62	3.12
Plate	0.01	0.00	0.12	4.05	0.00	0.01	0.01
Gender	− 0.03	0.02	− 0.04	− 1.44	0.15	− 0.08	0.01
Gestational age at birth	0.00	0.00	− 0.05	− 1.70	0.09	0.00	0.00
BMI	− 0.01	0.00	− 0.06	− 1.94	0.05	− 0.01	0.00
Smoking	0.04	0.05	0.02	0.76	0.44	− 0.06	0.14
BNSQ total score	0.00	0.00	− 0.02	− 0.59	0.56	− 0.01	0.01
BNSQ16	0.03	0.03	0.04	1.30	0.19	− 0.02	0.09
CESD	0.00	0.02	− 0.01	− 0.16	0.87	− 0.05	0.04
a. Dependent variable: LTL							

STAI = Short version of State and Trait anxiety Scale, Trait version; CES-D = Center for Epidemiological Studies Depression Scale; PSS = Perceived Stress Scale; BNSQ total score = Basic Nordic Sleep questionnaire total score;BNSQ 16 = Question “do you snore?” dichotomized as “yes” or “no”; BMI = Height in meters/weight^2^.

**Table 2:** Linear regression model with Leukocyte Telomere length as a dependent variable and anxiety (STAI); plate, child ‘s gender; maternal smoking; maternal BMI; gestational age at birth; and maternal BNSQ total score and snoring (BNSQ16) as explanatory variables.

Unstandardized coefficients	Standardized coefficients			t	Sig	95% confidence interval for B	
	B	SE	Beta			Lower bound	Upper bound
(Constant)	2.51	0.39		6.50	0.00	1.75	3.27
Plate	0.01	0.00	0.11	3.92	0.00	0.00	0.01
Gender	− 0.03	0.02	− 0.03	− 1.21	0.23	− 0.07	0.02
Gestational age at birth	0.00	0.00	− 0.05	− 1.61	0.11	0.00	0.00
BMI	− 0.01	0.00	− 0.06	− 2.08	0.04	− 0.01	0.00
Smoking	0.03	0.05	0.02	0.59	0.55	− 0.07	0.12
BNSQ total score	0.00	0.00	− 0.01	− 0.19	0.85	− 0.01	0.01
BNSQ16	0.04	0.03	0.04	1.51	0.13	− 0.01	0.09
STAI	− 0.09	0.05	− 0.06	− 2.05	0.04	− 0.18	0.00
a. Dependent variable: LTL							

STAI = Short version of State and Trait anxiety Scale, Trait version; CES-D = Center for Epidemiological Studies Depression Scale; PSS = Perceived Stress Scale; BNSQ total score = Basic Nordic Sleep questionnaire total score;BNSQ 16 = Question “do you snore?” dichotomized as “yes” or “no”; BMI = Height in meters/weight^2^ .

**Table 3:** Linear regression model with Leukocyte Telomere length as a dependent variable and stress (PSS); plate; child ‘s gender; maternal smoking; maternal BMI; gestational age at birth; and maternal BNSQ total score and snoring (BNSQ16) as explanatory variables.

	Unstandardized coefficients	Standardized coefficients			t	Sig	95% confidence interval for B	
		B	SE	Beta			Lower bound	Upper bound
1	(Constant)	2.34	0.38		6.19	0.00	1.60	3.08
	Plate	0.01	0.00	0.11	3.90	0.00	0.00	0.01
	Gender	− 0.03	0.02	− 0.03	− 1.19	0.24	− 0.07	0.02
	Gestational age at birth	0.00	0.00	− 0.05	− 1.61	0.11	0.00	0.00
	BMI	− 0.01	0.00	− 0.06	− 2.00	0.05	− 0.01	0.00
	Smoking	0.03	0.05	0.02	0.57	0.57	− 0.07	0.12
	BNSQ total score	0.00	0.00	− 0.02	− 0.72	0.47	− 0.01	0.00
	BNSQ16	0.04	0.03	0.04	1.43	0.15	− 0.01	0.09
	PSS	0.00	0.00	− 0.01	− 0.19	0.85	− 0.01	0.01
a. Dependent Variable: LTL								

STAI = Short version of State and Trait anxiety Scale,Trait version; CES-D = Center for Epidemiological Studies Depression Scale; PSS = Perceived Stress Scale; BNSQ total score = Basic Nordic Sleep questionnaire total score;BNSQ 16 = Question “do you snore?” dichotomized as “yes” or “no”; BMI = Height in meters/weight^2^ .

## Discussion

We found no associations between the quality of mothers’ sleep during pregnancy and LTL of newborn children, as measured from blood leukocytes sampled at birth. We were also unable to replicate previous findings related to maternal wellbeing during pregnancy and newborn LTL, despite our sample size outnumbering most previous studies. Increased maternal anxiety, as measured with a standard questionnaire during the last trimester (STAI), was associated with shorter LTL, but this result did not remain significant after correcting for multiple testing. Associations between maternal wellbeing and newborn telomere length should be carefully considered, and there may be multiple reasons for the discrepancies between our results and those previously reported.

First, differences in the LTL analysis method may be a source of undesired variation and thus make it difficult to replicate findings. Most studies used quantitative real-time PCR as we did, but some also used a restriction-enzyme–based approach. Some studies performed analysis in triplicates, while others performed duplicates and some did not clearly report this phase of the analysis. Again, even using the same kind of methodology, we could not replicate previous findings on the effect of maternal stress during pregnancy^22^ .The qPCR method is prone to large between-sample variation and measurement error, which can obscure patterns even when between-plate variation in T/S is taken into account. Furthermore, maternal effects on newborn LTL may also manifest as a shift in the distribution of long versus short telomeres^24^; if this was the case, a method based on measuring the average relative LTL would not necessarily detect these effects.

Second, the statistical models used in the analyses varied between studies. Most studies used maternal-related covariates such as maternal age, maternal BMI, or smoking. Some studies also used pregnancy-related covariates such as diabetes, preeclampsia, or number of previous pregnancies. When using a linear model with multiple explanatory factors that can be causative for each other, a collider bias might exist^35^. For example, maternal high BMI might have an effect on child’s LTL^26^ as well as maternal diabetes^30^. However, high BMI can be partially causative for diabetes, introducing a colliding effect. Furthermore, many studies used child-related covariates such as gender, ethnicity, birth weight, and gestational age. The statistical model used most often was a linear model constructed from different sets of the aforementioned factors. Other models were also used, such as arranging weight gain into groups^29^. Despite using the same kind of model as previously reported^22^ with only Perceived stress scale (PSS) as a predictor of LTL, we were unable to replicate findings related to PSS (β − 0.00, *p* = 0.661), Supplement S1.

A third source of variation may be the different cell populations present in umbilical blood samples. If different leukocyte types have different TL, differences among individuals might not reflect true differences in LTL as much as differences in distributions of types of leukocytes among individuals^36^. This so-called “tissue issue” is familiar in the field of epigenetics, particularly in methylation studies, where good algorithms have been developed to adjust or control for this issue^37^. In LTL studies, however, such an algorithm would not be applicable and ways to control differences in white-blood-cell populations between samples are not typically used. When TL measured in white blood cells collected from umbilical cord samples were compared with TL measured from placental tissue cells, there were no differences in TL between different tissues of origin^27^. In our sample (n = 1405) we could hypothesize that sample size is sufficient such that the cell populations among samples would be normally distributed making this an unlikely source of bias in our findings. However, typical sample sizes in previous studies vary, from a few dozen to a few hundred individuals, and only two previous studies^22,27^ had sample sizes over three hundred. Therefore, one might not automatically expect a normal and even distribution of white-blood-cell types across individual samples within a small sample size.

Finally, measures of maternal wellbeing and the timing of measurements varied between studies. In this study, maternal predictors (phenotypes) were collected using partly different questionnaires than in previous studies. As different questionnaires might have different psychometric properties, such as sensitivity and specificity, they can partially measure different phenomena. In previous studies, questionnaires were used as either continuous or dichotomized, yielding slightly different results. Furthermore, cut-off points for dichotomizing varied, as well as the timing of questionnaires during pregnancy. Yet, we could not replicate previous findings even when we used the same well-validated questionnaire (PSS) in the same way and in the same trimester as in a prior study^22^. Another line of evidence that links maternal wellbeing to offspring LTL comes from studies assessing maternal stress through physiological measures, such as hair cortisol. Circulating glucocorticoids are a potential mechanism of telomere attrition, but the relationship between experienced stress and cortisol output depends on many other factors, such as individual coping style^38^. One possible mechanism behind the fetal programming of telomere biology-hypothesis^1^ is the effect of the maternal HPA axis on newborns’ telomeres. Indeed, stress can have a shortening effect on LTL via the HPA-axis^39^. This effect coud be modified during pregnancy, however, by both fetal gender-related specific effects on maternal cortisol levels, and a a gender-specific effect of maternal cortisol on newborns’ LTL^40^. In addition to fetal gender, many lifestyle, socioeconomic, and biological factors—such as maternal smoking, being unemployed, BMI, c-reactive protein, and gestational age, as well as sampling time—were associated with maternal cortisol levels during pregnancy^41^. Psychosocial stress, however, had no effect on maternal cortisol levels during pregnancy. Another study found contrary results indicating an effect of maternal psychosocial stress on cortisol levels during pregnancy^42^. Besides cortisol, other mechanisms such as TNFα-mediated shortening of telomeres have also been reported^43^. Careful consideration regarding consistency and the relationship between different measures of maternal wellbeing is therefore needed. For example, trajectories af maternal wellbeing during pregnancy^44^ could be associated with outcomes, instead of relying on questionnaire items at single timepoints, or multiple analyses using correlated predictors.

Many of the aforementioned aspects that may cause heterogenity between studies have been identified in a recent large meta-analysis of early adversities and LTL^45^. It is worth noting that of 41 studies included in this meta-analysis, only two used umbilical-cord blood samples as a source of tissue and thus no definite conclusions could be drawn relative to newborns without additional data.

There are several strengths in our study: Our sample size is large, the study is population-based, and samples are collected from a defined geographical region. All measurements of maternal wellbeing were conducted at the same timepoint during the third trimester, with well-validated questionnaires^46^. In addition, all measurements of LTL were performed in the same laboratory, at the same time, ensuring a high quality of analysis.

Some weaknesses do appear. Due to the fact that this is a population-based sample, only a minority of subjects suffered from severe stress, anxiety, or clinically significant depression, which could be expected to affect the child through fetal programming^1^. Self-reported stress during pregnancy was low in our sample—the mean score for PSS was 5.67 (SD = 2.83). In a clinical or high-risk sample, associations absent from this study might be detectable. For example, smoking had no effect on newborns’ LTL, similar to a previous study by Send et al.^22^. Smoking during pregnancy is quite rare in population-based samples; in our sample, only 5.7% of all mothers reported smoking during pregnancy. We did not observe an effect of maternal sleep on newborn LTL. This might suggest that sleep has no effect, or that different measurements of sleep are required to detect such an effect. In our population-based sample, the number of cases with severe sleep disturbances was small; it is possible that an effect would be detectable in case–control studies.

In conclusion, there may be multiple reasons for the diversity of findings observed concerning newborn LTL and various maternal-related factors, such as stress during pregnancy, anxiety, depression, or pre-pregnancy BMI. As solid evidence exists that these factors do have an effect on a child’s development^47–49^, research into the possible molecular mechanisms is needed. Although LTL might serve as a marker for a good outcome measurement for these factors, methodological considerations should be vigorous in order to improve the reproducibility of findings. A publication bias might also exist, as there are no published negative findings on newborn LTL and various maternal and pregnancy-related topics.

## Materials and methods

### Samples

We used a large prospective Finnish birth cohort—the CHILD-SLEEP (CS) cohort^46^. A prenatal questionnaire on sleep quality, stress, depression, and anxiety was administered during the third trimester (gestation week 32).

The CS cohort was recruited from the Pirkanmaa Hospital District in Finland. Ethical approval was granted from the Pirkanmaa Hospital District ethical committee. All methods were performed in accordance with the relevant guidelines and regulations, and written informed consent was obtained from all participants. Parent’s informed consent was obtained for newborns. All children were born between 2011 and 2013. LTL was measured from a total of 1405 newborns (91.8% of all children with DNA available). The characteristics of our sample are described in Table 4. Children were born by spontaneous vaginal birth in 82.4% of cases, with vacuum-assisted vaginal birth in 7.5%, by elective cesarean section in 2.9%, and by acute cesarean section in 7.2%. The following variables were calculated from the study questionnaires: *Maternal stress* during pregnancy was evaluated with the short version of the Perceived Stress Scale (PSS)^50^. The scale measures perceived stress on a five-point scale. Summary scores ranged from 0 to 17 and Cronbach’s α for total score was 0.67. *Maternal depression* during pregnancy was evaluated with the Center for Epidemiological Studies Depression Scale (CES-D)^51^. The range of total scores was 2–23 (Cronbach’s α 0.78). *Maternal anxiety* during pregnancy was measured with the Short version of State and Trait anxiety Scale, Trait version (STAI)^52^. Total scores ranged from 6 to 21; Cronbach’s α was satisfying (0.78). *Sleep quality* was evaluated with The Basic Nordic Sleep questionnaire (BNSQ)^53^. The questionnaire has 21 items that measure self-reported sleep quality, sleep latency, sleep duration, and tiredness, among other measures. We calculated the insomnia score from BNSQ by summing up insomnia-related questions (BNSQ 1, 3, 4, 5, and 6) into a total insomnia score. This score ranged from 5 to 25 (Cronbach’s α 0.70). Snoring was dichotomized from BNSQ question 16 (“Do you snore?”); snoring one to two times a week or more was considered as a case. Smoking was evaluated with the question “Have you smoked during last 6 months?”; those who provided a “yes” answer were considered a smoker. Pre-pregnancy BMI was calculated from self-reported weight divided by self-reported height^2^ .

**Table 4:** Characteristics of study sample.

Continous variables	Mean	SD	n
Maternal age	30.64	4.57	1323
Maternal BMI (kg/m^2^)	28.43	4.42	1351
Anxiety (STAI)	8.91	2.37	1390
Depression (CES-D)	7.01	3.42	1348
Stress (PSS)	5.67	2.83	1377
BNSQ insomnia score	14.80	3.31	1393
Child´s gestation age at birth, days	280.70	8.51	1340
Birth weight (g)	3597	449	1414
Birth weight 25% percentile( g)	3295		
Birth weight 50% percentile (g)	3580		
Birth weight 75% percentile(g)	3880		
Apgar score 5 min	8.64	1.40	1337

Dichotomous variables	n (cases) %	n (controls) %	Total
Maternal Snoring (BNSQ16)	330 (25%)	991 (75%)	1321
Maternal smoking	79 (5.7%)	1308 (94.3%)	1387
Child´s gender	M = 693 (51.7%)	F = 647 (48.3%)	1340

BMI = Body Mass Index, self-reported weight divided by self-reported height^2^; STAI = Short version of State and Trait anxiety Scale,Trait version; PSS = Perceived Stress Scale; CES-D = Center for Epidemiological Studies Depression Scale;Apgar = 5-min Apgar score; BNSQ 16 = Question “do you snore?” dichotomized as “yes” or “no”.

### Power analysis

We performed power calculations based on the literature to explore whether our sample was sufficient to replicate previous findings regarding pregnancy-related maternal variables and their association with newborn LTL. We selected maternal stress, BMI, smoking during pregnancy, depression, and anxiety during pregnancy as variables of interest. As there were no previous studies regarding sleep quality on LTL, we used sleep apnea in adults as a source for effect size. We extracted the effect size and error level from selected publications (Table 5). From our analyses, the power varies from 0.89 to 0.999.

**Table 5:** Power analysis.

Variable	Publication	Effect size from reference publication	Power (n)	Minimun effect size detectable with 80% power
Body mass index BMI	Martens DS, Plusquin M, Gyselaers W, et.al: Maternal pre-pregnancy body mass index and newborn telomere length. BMC Med. 2016 Oct 18;14(1):148 ^27^	r = − 0.11	0.999 (1351)	0.07
Smoking	Bosquet E M, Bollati V, Sideridis G, et.al: Sex differences in effects of maternal risk and protective factors in childhood and pregnancy on newborn telomere length. Psychoneuroendocrinology. 2018 Sep;95:74–85 ^23^	r = − 0.18	0.999 (1387)	0.07
Depression	Bosquet E M, Bollati V, Sideridis G, et.al: Sex differences in effects of maternal risk and protective factors in childhood and pregnancy on newborn telomere length. Psychoneuroendocrinology. 2018 Sep;95:74–85 ^26^	r = 0.24	1 (1348)	0.07
Sleep	Salihu HM, King L, Patel P et. al: Association between maternal symptoms of sleep disordered breathing and fetal telomere length. Sleep. 2015 Apr 1;38(4):559–66 ^32^	r = 0.375	= 1 (1392)	0.07
Stress	Send TS, Gilles M, Codd V, et. al: Telomere Length in Newborns is Related to Maternal Stress During Pregnancy. Neuropsychopharmacology. 2017 Nov;42(12):2407–2413 ^22^	r = 0.14	0.999 (1392)	0.07

Power refers to the calculated probability to detect a given effect with a given sample size, e.g. it describes the changes to find an effect previously found with a given sample size.

### Measurement of leukocyte telomere length

LTL was measured from DNA extracted from peripheral blood taken from the umbilical cord at birth. DNA extraction from K2-EDTA-blood tubes was performed using magnetic bead technology with a PerkinElmer chemagic 360 instrument (Waltham, MA.USA) and CMG-704 kit.

DNA was eluted in 400 µl 10 mM Tris–EDTA elution buffer (PerkinElmer). DNA concentration was measured with a Quant-iT™ PicoGreen™ dsDNA Assay Kit. Samples were aliquoted with Tecan Genesis/Tecan Freedom Evo (Tecan Trading AG, Switzerland) and shipped on dry ice for genetic analyses. DNA extraction was performed at the THL Biobank.

To measure LTL, we used a qPCR-based method as described previously^54–57^. We used β-hemoglobin (Hgb) as a single-copy reference gene. Separate reactions for telomere and Hgb reaction were performed in paired 384-well plates, in which matched sample well positions were used. Ten nanograms of DNA were used for each reaction, performed in triplicate. Every plate included a seven-point standard curve, which was used to create a standard curve and to perform absolute quantification of each sample. Samples and standard dilutions were transferred into the plates using a multichannel pipet and dried overnight at room temperature. A specific reaction mix for the telomere reaction included 270 nM tel1b primer (5′-CGGTTT(GTTTGG)5GTT-3′) and 900 nM tel2b primer (5′-GGCTTG(CCTTAC)5CCT-3′), 0.2X SyBr Green I (Invitrogen, Carlsbad, CA.USA), 5 mM DTT (Sigma-Aldrich Saint-Louis MI, USA), 1% DMSO (Sigma-Aldrich), 0.2 mM of each dNTP (Fermentas, Waltham, MA.USA), and 1.25 U AmpliTaq Gold DNA polymerase (Applied Biosystems, Waltham, MA.USA) in a total volume of 15 µl AmpliTaq Gold Buffer II supplemented with 1.5 mM MgCl_2_. The Hgb reaction mix included 300 nM Hgb1 primer (5′-GCTTCTGACACAACTGTGTTCACTAGC-3′) and Hgb2 primer (5′-CACCAACTTCATCCACGTTCACC-3′) in a total volume of 15 µl of iQ SyBrGreen supermix (BioRad). The cycling conditions for telomere amplification were 10 min at 95 °C, followed by 25 cycles at 95 °C for 15 s and 54 °C for 2 min with signal acquisition. The cycling conditions for Hgb amplification were 95 °C for 10 min, followed by 35 cycles at 95 °C for 15 s, 58 °C for 20 s, and 72 °C for 20 s with signal acquisition. Reactions were performed with a CFX384 Real-Time PCR Detection System (Bio-Rad Hercules, Ca., USA). Melt-curve analysis was performed at the end of the run to ensure specific primer binding.

We used the Bio-Rad CFX Manager software to perform quality control. Samples with a standard deviation of more than 0.5 between triplicates were omitted from analysis. Plate effect was considered by analyzing five genomic DNA control samples on every plate. We normalized the telomere and Hgb signal values separately to the mean of these control samples before taking the T/S ratio. The control samples were used for calculating the coefficient of variation (CV) values, which was 8.42%. Samples with Z-scores < − 3.0 or > 3.0 were removed as outliers. From 1421 samples, 16 (1.1% of total sample) were discarded as outliers, and thus a total of 1405 samples were used.

### Statistical analyses

We used linear regression model with Leukocyte Telomere length as a dependent variable and maternal anxiety, depression, stress, BMI, Sleep, qPCR analysis plate, maternal smoking, and childs’ gestational age at birth and child’s gender as explanatory variables. Due to high intercorrelation between PSS, CESD, and STAI, they were analysed separately in order to avoid collinearity bias (correlation coefficients between 0.57 and 0.61, *p* < 0.001). CESD and STAI scores were ln transformated in order to obtain better fit for linear regression. All analyses were performed using SPSS v.24 IBM, Armonk, NY, USA.

Power analysis was performed using R software (R ≥ 3.5.1) with specific algorithms implemented in R package “pwr”^58^ and for FDR correction we used R-package “p.adjust.”^59^. We took effect-size estimates from reference publications^22,26,27,32^ and set the α error level to < 0.05. We also calculated minimum effect size detectable with 80% likelihood (α = 5%) using otherwise the same parameters.

## Supplementary information

Supplementary information.

## Data Availability

The datasets analysed during the current study are available from the corresponding author on reasonable request.
