# Supplementary material for: Maternal stress or sleep during pregnancy are not reflected on telomere length of newborns
Source: Sci Rep. 2020 Aug 19;10:13986. doi: 10.1038/s41598-020-71000-2 (PMC7438332; doi:10.1038/s41598-020-71000-2)
Supplement: Supplementary file 1 — Supplementary information. [file 41598_2020_71000_MOESM1_ESM.pdf]

Maternal Stress or Sleep During Pregnancy Are Not Reflected In Telomere Length of Newborns

Antti-Jussi Ämmälä*[1,2] , Emma I.K. Vitikainen[3] , Iiris Hovatta[4,5,6], Juulia Paavonen [7,8], Outi Saarenpää-Heikkilä [9,10], Anneli Kylliäinen [11], Pirjo Pölkki [12], Tarja Porkka-Heiskanen [5], Tiina Paunio [1,2,5]

1 Department of Genetics and Biomarkers, National Institute for Health and Welfare, Helsinki, Finland

2 Department of Psychiatry, University of Helsinki and Helsinki University Hospital, Helsinki, Finland

3 Organismal and Evolutionary Biology Research Programme, Faculty of Biological and Environmental Sciences, University of Helsinki

4 Department of Psychology and Logopedics, University of Helsinki

5 SleepWell Research Program, Faculty of Medicine, University of Helsinki

6 Neuroscience Center, Helsinki Institute of Life Science HiLIFE, University of Helsinki

7 Department of Public Health Solutions, National Institute for Health and Welfare, Helsinki, Finland

8 Pediatric Research Center, Child Psychiatry, University of Helsinki and Helsinki University Hospital

9 Tampere University Hospital, Department of Paediatrics,Tampere, Finland

10 Tampere University, Tampere Centre for Child Health Research, Tampere, Finland

11 Psychology, Faculty of Social Sciences, Tampere University, Finland

12 Department of -Social Sciences, University of Eastern Finland, Kuopio

[*]Corresponding author: Antti-Jussi Ämmälä; National Institute for Health and Welfare; Mannerheimintie 166, P.O. 30, 00271 Helsinki Finland. Tel: +35829 524 5550 antti-jussi.ammala@helsinki.fi

Table S1 In Send et al[22] Table 5 p.2411 line 2) presents model with only PSS and newborn telomere length. Model details were not presented in the publication. The same model in our sample:

Coefficients[a]

Model				Standardized Coefficients<br>Beta	t	Sig.
1	(Constant)	1,701	0,023		74,857	0,000
	PSS	-0,002	0,004	-0,012	-0,440	0,660

a. Dependent Variable: Newborn LTL
